# Neurodegenerative pathologies associated with behavioral and psychological symptoms of dementia in a community-based autopsy cohort

**DOI:** 10.1186/s40478-023-01576-z

**Published:** 2023-06-02

**Authors:** Ruth S. Nelson, Erin L. Abner, Gregory A. Jicha, Frederick A. Schmitt, Jing Di, Donna M. Wilcock, Justin M. Barber, Linda J. Van Eldik, Yuriko Katsumata, David W. Fardo, Peter T. Nelson

**Affiliations:** 1 Emory University, Atlanta, GA USA; 2 Sanders-Brown Center on Aging, University of Kentucky, Lexington, KY USA; 3 Department of Epidemiology and Environmental Health, University of Kentucky, Lexington, KY USA; 4 Department of Neurology, University of Kentucky, Lexington, KY USA; 5 Department of Pathology and Laboratory Medicine, University of Kentucky, Lexington, KY USA; 6 Department of Physiology, University of Kentucky, Lexington, KY USA; 7 Department of Neuroscience, University of Kentucky, Lexington, KY USA; 8 Department of Biostatistics, University of Kentucky, Lexington, KY USA; 9 University of Kentucky, Rm 575 Todd Building, Lexington, KY 40536 USA

**Keywords:** Synuclein, Tau, Psychiatric, Psychosis, Psychoses, FTLD, FTD, Aphasia, Tauopathy, HS-aging

## Abstract

In addition to the memory disorders and global cognitive impairment that accompany neurodegenerative diseases, behavioral and psychological symptoms of dementia (BPSD) commonly impair quality of life and complicate clinical management. To investigate clinical-pathological correlations of BPSD, we analyzed data from autopsied participants from the community-based University of Kentucky Alzheimer’s Disease Research Center longitudinal cohort (n = 368 research volunteers met inclusion criteria, average age at death 85.4 years). Data assessing BPSD were obtained approximately annually, including parameters for agitation, anxiety, apathy, appetite problems, delusions, depression, disinhibition, hallucinations, motor disturbance, and irritability. Each BPSD was scored on a severity scale (0–3) via the Neuropsychiatric Inventory Questionnaire (NPI-Q). Further, Clinical Dementia Rating (CDR)-Global and -Language evaluations (also scored on 0–3 scales) were used to indicate the degree of global cognitive and language impairment. The NPI-Q and CDR ratings were correlated with neuropathology findings at autopsy: Alzheimer’s disease neuropathological changes (ADNC), neocortical and amygdala-only Lewy bodies (LBs), limbic predominant age-related TDP-43 encephalopathy neuropathologic changes (LATE-NC), primary age-related tauopathy (PART), hippocampal sclerosis, and cerebrovascular pathologies. Combinations of pathologies included the quadruple misfolding proteinopathy (QMP) phenotype with co-occurring ADNC, neocortical LBs, and LATE-NC. Statistical models were used to estimate the associations between BPSD subtypes and pathologic patterns. Individuals with severe ADNC (particularly those with Braak NFT stage VI) had more BPSD, and the QMP phenotype was associated with the highest mean number of BPSD symptoms: > 8 different BPSD subtypes per individual. Disinhibition and language problems were common in persons with severe ADNC but were not specific to any pathology. “Pure” LATE-NC was associated with global cognitive impairment, apathy, and motor disturbance, but again, these were not specific associations. In summary, Braak NFT stage VI ADNC was strongly associated with BPSD, but no tested BPSD subtype was a robust indicator of any particular “pure” or mixed pathological combination.

**Supplementary Information:**

The online version contains supplementary material available at 10.1186/s40478-023-01576-z.

## Introduction

The clinical syndrome of dementia is characterized by impaired cognition and decreased ability to perform normal activities of daily living [100]. Beyond those cardinal clinical features, additional behavioral and psychological symptoms of dementia (BPSD) often cause distress for patients and caregivers, reduce quality of life, and predispose patients to institutionalization [24, 93, 112]. BPSD encompass a broad range of symptomatic domains that include linguistic, autonomic, and/or motor disturbance, in addition to new-onset neuropsychiatric disease symptoms. Relating specific BPSD subtypes to the neuropathologic changes that underly them is complicated by the diversity and dynamic nature of BPSD subtypes and also by the fact that multiple co-existing brain pathologies are very common. Previously published clinical-pathological association studies have indicated a generally additive impact of different pathologic subtypes on BPSD severity [14, 33, 42, 61, 66, 72, 75, 76, 79, 86, 91, 92, 101, 122, 125]. However, the associations between specific combinations of brain pathologies and a broad spectrum of BPSD subtypes are incompletely characterized. A better understanding of the relevance of specific symptoms to their underlying pathologic substrates may aid clinical trial stratification and other dementia-related research efforts.

Among persons aged 80 years or older, the most common and impactful neurodegenerative disease pathologies are (in the order of their estimated attributable risk to the Alzheimer’s-type dementia syndrome [14, 83]): Alzheimer’s disease neuropathologic changes (ADNC) [73], limbic predominant age-related TDP-43 encephalopathy neuropathologic changes (LATE-NC) [83], and Lewy body pathologies (LBP) [4]. In ~ 20% of older persons with dementia, all three neurodegenerative disease pathologies are present; this has been termed the quadruple misfolding proteinopathy (QMP) because Aβ, tau, α-synuclein and TDP-43 pathologies are present [46, 62, 103]. Among the pathologic phenotypes, there appear to be both biologic synergies (where the presence of one pathologic subtype alters the likelihood of another being present) [50, 110] and pleiotropic genetic risk factors [20]—for example, the same *APOE* ε4 allele that is associated with increased ADNC risk is also associated with increased risk for both LATE-NC and LBP [48, 118, 123]. Vascular pathologies also have a large impact on cognitive status and BPSD [24, 45, 113, 119].

Prior studies established that mixed pathologies are associated with altered clinical phenotypes, in comparison with pure pathologic patterns. A rapidly expanding literature supports the concept of additive (but not necessarily synergistic) impact on cognitive status [1, 2, 13, 18, 34, 37]. Thus, for a given severity of ADNC, the presence of LBP or LATE-NC in a brain is associated with more impaired global cognition than ADNC alone [79, 86]. As one might expect, the QMP phenotype is associated with relatively severe dementia [52, 53].

In addition to the clinical syndrome of amnestic dementia, the presence of mixed pathologies has also been associated with increased risk for a diverse range of symptoms, i.e., BPSD [30, 31, 63, 106, 119]. Some specific BPSD symptoms were linked to particular pathologic patterns in prior work. For example, LBP has been associated with autonomic and movement disorders (parkinsonism), and hallucinations [67, 68, 71]. Other neuropsychiatric symptoms are well known to be experienced by patients with ADNC—apathy and irritability, for example. There are also important unanswered questions, such as whether aging-related TDP-43 proteinopathy (i.e., LATE-NC) manifests clinically with the distinctive symptoms of frontotemporal dementia (FTD), such as disinhibition and aphasia [27, 51, 54, 60, 97].

To elucidate relationships between multiple BPSD and common underlying neuropathologic patterns, we analyzed data from the University of Kentucky Alzheimer’s Disease Research Center (UK-ADRC) autopsy cohort. The UK-ADRC autopsy cohort draws from a community-based group of research volunteers who were mostly recruited while cognitively normal and followed with approximately yearly clinic visits – often for over a decade [102]. Our goals were to identify neuropathologies that underlie different BPSD subtypes and to estimate the association of individual BPSD symptoms with specific subsets of neuropathologies.

## Methods

### Participants

The UK-ADRC autopsy cohort, a community-based cohort actively recruiting from the Lexington, Kentucky region, was described previously along with recruitment details [84, 102, 110]. Briefly, older adult volunteers agreed to be followed annually for cognitive, physical, and neurological examination and to donate their brain at the time of death. Protocols were approved by the University of Kentucky Institutional Review Board, and all participants provided written informed consent. Certain exclusion criteria—including parkinsonism, active substance use disorder, and severe neuropsychiatric disorder (e.g. bipolar disorder or schizophrenia)—were applied prior to recruitment [102, 110], but participants who developed these conditions while in the study were not excluded.

For a participant’s data to be included in the current study, we required availability of replete ADNC, LBP, TDP-43 proteinopathy data, and availability of BPSD data (see below), with 392 individuals meeting these inclusion criteria. Routine neuropathological assessments of these conditions began in 2012, so as a result the included cases were autopsied from 2012 to 2022. Following the application of these criteria, we further excluded autopsied participants with rare diseases (e.g., prion disease, frontotemporal lobar degeneration [FTLD], triplet repeat disorders) who were recruited directly from a University of Kentucky memory disorders clinic (n = 24 additional exclusions), for a final n = 368 participants included.

### Assessment of BPSD and dementia severity

Beginning in 2005, UK-ADRC implemented the data collection protocol defined by the National Alzheimer’s Coordinating Center (NACC) Uniform Data Set (UDS), which is a standardized data collection protocol used by all National Institute on Aging-funded ADRCs [7]. One of the UDS instruments is the Neuropsychiatric Inventory Questionnaire (NPI-Q) [55]; this instrument corresponds to UDS Form B5 https://files.alz.washington.edu/documentation/uds3-tip-b5.pdf), which is used to assess the presence and severity of specific BPSD experienced by each participant, as rated by a reliable study partner. BPSD symptoms assessed in the NPI-Q (scored on a 0–3 semiquantitative scale) include: agitation, anxiety, apathy, appetite problems, delusions, depression, disinhibition, motor disturbances, hallucinations, and irritability. Although assessed on the NPI-Q questionnaire, elation and night-time behaviors were not included in the present study due to too few endorsements of these parameters. Participants and their study partners also were administered the Clinical Dementia Rating (CDR; UDS Form B4 https://files.alz.washington.edu/documentation/uds3-ivp-b4.pdf) [74] at each visit [59]. We note that the NPI-Q and CDR are completed for all ADRC participants, not only those with dementia. For an overview of the operationalizations of BPSD subtypes and the criteria used for the NPI-Q and CDR instruments, see Additional file 1: Table S1.

### Neuropathologic assessment

Detailed protocols for the neuropathologic workup at the UK-ADRC were previously described [3]. Neuropathologic endpoints were characterized using conventional neuropathologic diagnostic methodologies. Aβ plaques were detected with Nab228 antibody (gift from Dr. Eddie Lee); tauopathy was detected using the PHF-1 phospho-Tau (pSer396) antibody (gift from Dr. Peter Davies); TDP-43 proteinopathy with the 1D3 phospho-TDP-43 (pSer409/pSer410) antibody (BioLegend, Inc., San Diego, CA); and, Lewy bodies (LBs) with anti-α-Synuclein KM51 antibody (Leica Biosystems, Inc., Buffalo Grove, IL). Using these reagents and methods described previously [52, 81, 90], we scored consensus-based and conventional neuropathologic endpoints, including hippocampal sclerosis (HS) [73], Braak neurofibrillary tangles (NFT) stages [15], Thal Aβ phases [116], and LATE-NC stages [83, 88]. For LBP [4], all cases were screened using the anti-α-Synuclein antibody in the olfactory bulb, amygdala, medulla, midbrain, and basal ganglia, with neocortical regions (mid-frontal gyrus, inferior parietal lobule, superior and middle temporal gyrus, temporal pole) assessed for cases with any LBP in the screening slides. For operationalization of primary age-related tauopathy (PART) [22], we evaluated subjects with CERAD neuritic amyloid plaque levels [69] of “none”. We also included assessments for cerebrovascular disease, operationalized using scored parameters for multiple pathologies (Circle of Willis atherosclerosis, arteriolosclerosis, cerebral amyloid angiopathy [CAA], microinfarcts, lacunes, and gross infarcts) [109].

A key goal of the present work was to include analyses of mixed pathologies, and to test their association with BPSDs. Even without factoring in cerebrovascular pathologies, there are seven different potential combinations of prevalent pathologic phenotypes: Pure ADNC, Pure LATE-NC, Pure LBP, ADNC + LATE-NC, ADNC + LBP, LATE-NC + LBP, and ADNC + LATE-NC + LBP. Given the complexity of the neuropathologic phenotypes, and the lack of a universal and/or consensus-based method to categorize mixed pathologies, ad hoc thresholds were applied to characterize each phenotype as absent or present. These categories were generated a priori and not changed thereafter. Criteria that were applied reflect the pathologic severities associated with neurological impairments. For the presence of ADNC: Braak NFT stages V or VI with any detected cerebral neuritic amyloid plaques (operationalized with CERAD criteria [69]); for the presence of LATE-NC: LATE-NC stages 2–3 [83]; and, for the presence of LBP: any detected neocortical LBs. We also applied a diagnostic category of vascular pathology to summarize the cerebrovascular pathologies listed above, which was generated separately for each case independently of the present study as part of routine assessments, to convey that the burden of large and small infarcts, and small vessel disease, were likely to collectively or individually contribute to the cognitive impairment.

### Statistical analyses

To evaluate the relationships between BPSD subtypes and the various neuropathologic groupings, we employed a case–control design, where participants with neuropathologic phenotypes were the cases and those lacking severe neurodegenerative neuropathologies (i.e., lacking Braak NFT stages > IV, LATE-NC stages > 1, or neocortical LBs) were controls. The severity of NPI-Q items was operationalized based on the highest reported severity for each symptom, across their annual UDS assessments, as symptoms are dynamic and may improve or worsen over time; the NPI-Q measures symptoms that occurred only within the month prior to the study visit. For example, if an individual had severe (score = 3) agitation three years before death, but her final exam was only mild (score = 1), then her reference number for agitation would still be 3 for the sake of the current analyses. Further, some of the NPI-Q BPSD subtype descriptions are aimed more at Alzheimer’s-type clinical disease rather than other subtypes of symptomatic manifestations. As such, the “motor disturbance” cued by the NPI-Q assessment prompt focuses on repetitive motoric behaviors (“…pacing around the house…”), rather than being related to motor features of parkinsonism (gait problems, rigidity, etc.). The CDR data were taken from the participant’s last assessment prior to death.

Unadjusted and age-adjusted mean numbers of BPSD were estimated across the groups. Age-adjustment was implemented via Poisson regression models using the glm() function in R [38, 114]. The predict() function yielded the adjusted mean BPSD counts for each pathological category, holding age at death at its overall mean.

**Fig. 1:**
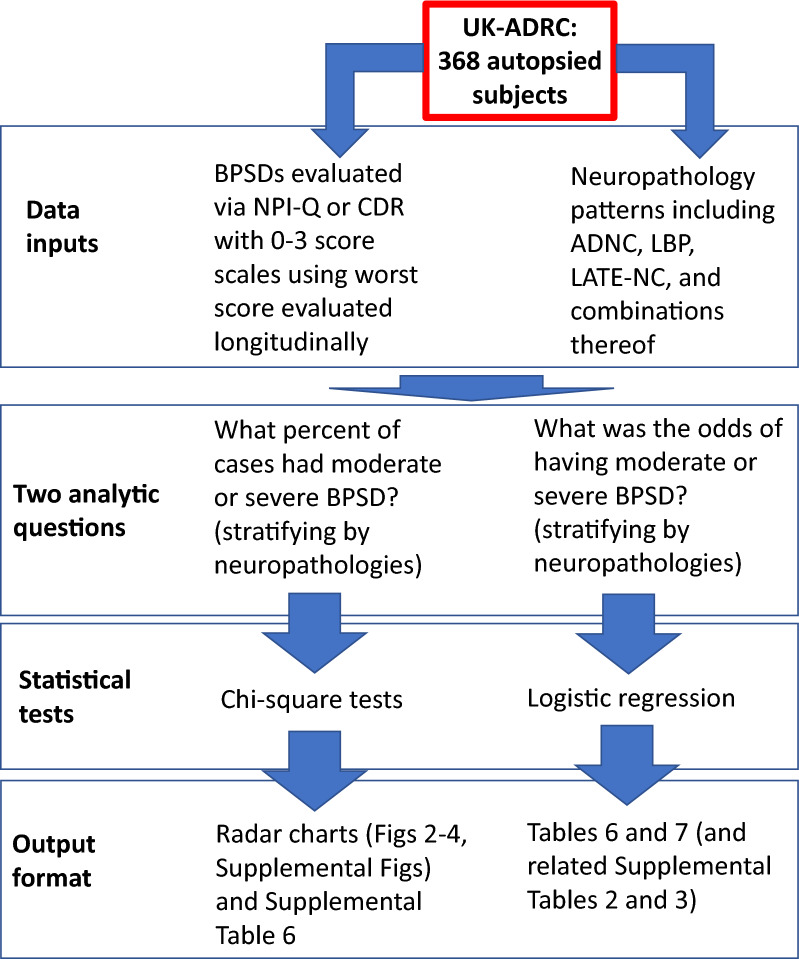
A schematic representation of selected data analyzed, questions addressed, and statistical analyses in the present study

Two additional questions were addressed using statistical tests, as shown in Fig. 1. To compare severity of BPSD within pathology groups, BPSD symptom ratings were dichotomized based on scores of 2 or 3 (moderate or severe) versus 0 or 1 (not present or mild). For each BPSD subtype, chi-square analyses were used to test whether moderate-to-severe BPSD was disproportionately distributed in each pathology group versus control. Radar charts were used to visualize the proportion of individuals in each specified pathological group who had moderate-to-severe symptoms for each BPSD. To perform covariate-adjusted analyses of severity, logistic regressions were used to estimate the association between each BPSD and each pathological group. For these analyses, we combined delusions and hallucinations into a single category of “psychoses”, i.e. if a subject had either moderate-to-severe delusions or moderate-to-severe hallucinations, he or she would be considered to have moderate-to-severe psychoses. Each model utilized a subset of the data containing the low-pathology controls and the pathological group of interest. Thus, we fit a series of binary logistic models to the data rather than multinomial logistic regression; each approach has strengths and weaknesses, and we selected the series of binary models due to sparse data in some cells [11]. Covariates age at death, sex, and presence of at least one *APOE* ε4 allele were included in the statistical model to estimate the adjusted odds of moderate-to-severe BPSD symptoms. Additional logistic regressions (e.g., testing associations with certain Braak NFT stages or additional pathologies such as HS) dichotomizing outcome variable BPSD severity as any (1, 2, or 3 scoring) as opposed none (0 scoring) were performed as sensitivity analyses. From these logistic regressions, odds ratios, 95% confidence intervals, and *p* values were extracted. All regressions were performed using the logit model in the glm function in R [38, 114].

## Results

Participant characteristics are shown in Tables 1 and 2. A total of 368 autopsied volunteers met the inclusion criteria; they were predominantly highly educated, with an average age at death of 85.4 years. Further, 54.2% were diagnosed with dementia prior to death, and 38.6% had at least one *APOE* ε4 allele. About 25% of included participants were cognitively normal in the final evaluation prior to death in this community-based sample.

**Table 1:** Average age at death, sex, interval between final clinic visit and death, and years followed on study among included participants (n = 368)

Variable	Average value
Age at Death (Mean years ± StDev)	84.4 ± 8.8
Sex (% Female)	57.10%
Interval between last clinic visit and death (Mean years ± StDev)	1.3 ± 1.2
Years of longitudinal follow up on study (Mean years ± StDev)	10.1 ± 6.8

**Table 2:** Select demographic, clinical, and genetic features of included participants (n = 368)

Variable	Group	Frequencies	%
*APOE*	No ε4 allele	222	61.4
	1 or 2 ε4 alleles	139	38.6
Total		361	
Clinical status before death	Normal	87	23.7
	MCI*	65	17.7
	Demented	199	54.2
	Impaired/other	16	4.4
Total		367	
Education (Years)	5–10	5	1.4
	11–15	118	32.2
	16–20	233	63.7
	21–30	10	2.8
Total		366	

*MCI Mild cognitive impairment

Summary data about the neuropathologies are depicted in Table 3. Almost 40% of included participants had severe ADNC (Braak NFT Stages V or VI), ~ 30% had LATE-NC Stage > 1, and ~ 18% had neocortical LBP. Although over half of the participants were diagnosed with dementia prior to death, only n = 76 (20.7% of the overall cohort) had pure ADNC with Braak NFT stages V or VI. Cerebrovascular pathologies were also quite frequent, and these were parsed as infarcts (large or lacunar), arteriolosclerosis, or cerebral amyloid amyloidosis (Table 3).

**Table 3:** Select neuropathologic features of included participants (n = 368 total)

Pathology	Group	%
Braak NFT* stages	0–II	37.5
	III–IV	17.0
	V–VI	45.5
Any Lewy bodies	None	62.0
	Any	38.0
Neocortical LBs* severity	0	81.8
	1	5.4
	2–3	12.7
LATE-NC* stages	0	55.4
	1	14.7
	2–3	29.9
Infarcts	None	54.1
	Any	45.9
Arteriolosclerosis severity	None/mild	62.8
	Moderate/severe	37.2
CAA* severity	None/mild	79.6
	Moderate/severe	20.4

*NFT Neurofibrillary tangle, *LBs* Lewy bodies, *LATE-NC* Limbic predominant age-related TDP-43 encephalopathy neuropathologic changes, *CAA* Cerebral amyloid angiopathy

To enable dichotomous parameters of neuropathologies, we applied neuropathologic cut-points that have been robustly associated with neurologic symptoms: Braak NFT stages > IV; neocortical LBs; and LATE-NC stages > 1 [82, 83, 85, 87]. These cut-points were the basis for subsequent clinical-pathological correlations and the rationale for them are presented in Table 4. Applying these parameters, sample sizes and mean age at death of participants categorized by neuropathologies are presented in Table 5. The control group that had relatively sparse pathology (lacked Braak NFT stages > IV, LATE-NC stages > 1, or neocortical LBs) comprised 136 participants.

**Table 4:** Criteria for pathologically-defined brain conditions using dichotomous operationalizations

Disease or condition	Pathological hallmark related most directly to impairment	Staging system	Threshold for dichotomous scoring	Rationale	References
Alzheimer’s disease (AD)	Severe ADNC: Tau NFTs	Braak NFT Staging	Different for different tests	Braak NFT stages V/VI indicate “severe” ADNC and are consistently associated with impairment	[47, 73, 82]
Primary age-related tauopathy (PART)	Non-FTD Tau NFTs without neuritic amyloid plaques	Braak NFT Staging, CERAD scoring of neuritic amyloid plaques	“Probable PART”: CERAD negative Braak NFT stage 0-II versus III/IV	Braak NFT Stages III/IV are associated with impairment; PART typically does not expand to merit the diagnosis of Braak NFT stages V or VI	[22, 80]
Limbic-predominant age-related TDP-43 encephalopathy (LATE)	TDP-43 pathology	LATE-NC Stages	LATE NC stages 0–1 versus 2–3	LATE-NC stages 2–3 are most robustly associated with cognitive impairment; LATE-NC stage 1 cases are often non-impaired	[83, 88]
Hippocampal sclerosis	Dropout of neurons in CA1 and subiculum of hippocampal formation with gliosis	No true consensus-based system	Already is a dichotomous variable	It has previously been shown that in cases with LATE-NC, HS pathology is associated with worse outcomes	[40, 89]
Lewy body disease (LBD)	Neocortical Lewy bodies	Attems et al. [4]	Presence of neocortical LBs	There is no perfect or consensus-based staging system to dichotomize LBD. There is subclinical disease in many cases	[4, 49]
Vascular pathologies	Vascular pathologies comprise a heterogeneous group of disorders including large, medium-sized, and small infarctions, cerebral amyloid angiopathy, atherosclerosis, arteriolosclerosis. This was not a central focus of the present article partly because there is imperfect consensus-based methods for clinical-pathological correlation. However, at the UK-ADRC, a judgment is made for every deceased subject, at a clinical-radiographical-pathological consensus, about whether cerebrovascular factors contributed strongly to the patient’s clinical syndrome				[109]

**Table 5:** Sample sizes and average age at death stratified by neuropathological groups for included subjects (n = 368)

Braak NFT stage > IV	LATE—NC (Stage > 1): No		LATE—NC (Stage > 1): Yes	
	Neocortical LBs No	Neocortical LBs Yes	Neocortical LBs No	Neocortical LBs Yes
Sample numbers (% of participants)				
No	136 (37.0%)	24 (6.5%)	34 (9.2%)	8 (2.2%)
Yes	76 (20.7%)	22 (6.0%)	54 (14.7%)	14 (3.8%)

Braak NFT stage > IV	LATE—NC (Stage > 1): No		LATE—NC (Stage > 1): Yes	
	Neocortical LBs No	Neocortical LBs Yes	Neocortical LBs No	Neocortical LBs Yes
Age at death: Years, Avg ± SD				
No	85.9 ± 0.6	86.6 ± 1.2	90.3 ± 1.4	90.8 ± 3.4
Yes	81.8 ± 1.2	78.8 ± 2.2	88.0 ± 0.9	83.8 ± 2.6

In the UK-ADRC community-based cohort, multiple BPSD subtypes were often present among included participants. Age-adjusted mean numbers of BPSD per individual are shown in Table 6, whereas unadjusted mean numbers of BPSD per individual are shown in Additional file 2: Table S2. Even among individuals lacking substantial neurodegenerative disease pathologies, an average of 3.2 BPSD subtypes per person was documented. On the other hand, for individuals with comorbid Braak NFT stages > IV, neocortical LBs, and LATE-NC stage > 1 (the QMP phenotype), the average number of different BPSD subtypes before death was 8.6 per participant (9.7 age-adjusted). More details on the numbers in each group stratified by BPSD values are shown in Additional file 2: Table S3.

**Table 6:** Average number of different BPSD subtypes^a^ per individual participant by pathology category (Age-adjusted^b^)

Braak NFT stage > IV	LATE—NC^c^ (stage > 1): No		LATE—NC (stage > 1): Yes	
	Neocortical LBs^c^ No	Neocortical LBs Yes	Neocortical LBs No	Neocortical LBs Yes
No	3.6	4.1	4.9	7.8
Yes	6.4	7.6	7.5	9.7

*LBs* Lewy bodies

^a^BPSD subtypes included were agitation, anxiety, apathy, appetite problems, delusions, depression, disinhibition, motor disturbances, hallucinations, and irritability (by NPI-Q), and language disturbance (CDR-Language); see Additional file 1: Table S1. Parkinsonism was not analyzed as a BPSD subtype

^b^For raw mean numbers of BPSD per individual, see Additional file 2: Table S2

^c^LATE-NC = limbic predominant age-related TDP-43 encephalopathy neuropathologic changes

**Fig. 2:**
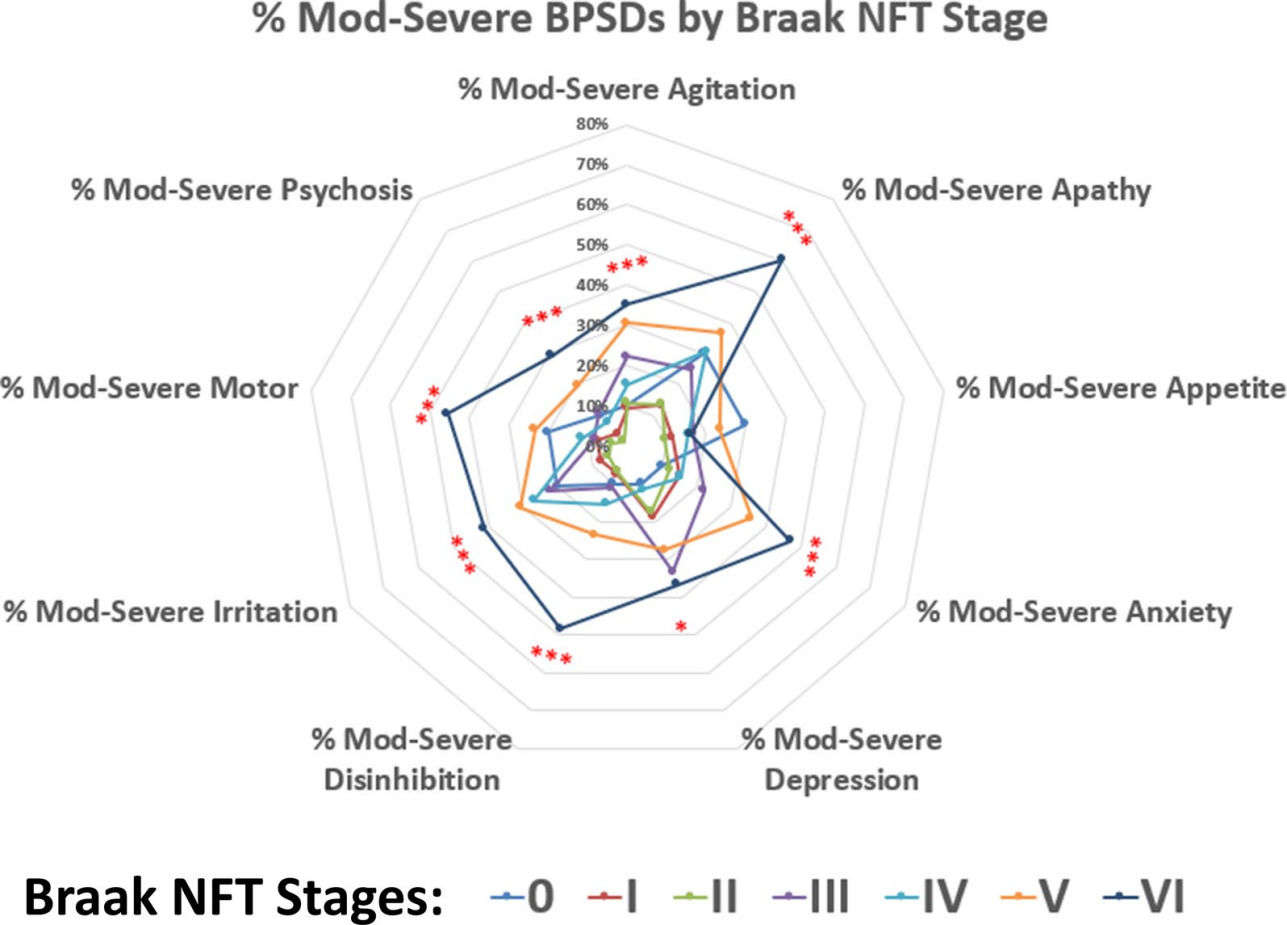
Radar chart depicts the percent of cases with moderate or severe BPSD subtypes, stratified by Braak NFT stages (0-VI). Cases included for this chart were selected among participants (n = 212) that lacked neocortical LBs and also lacked LATE-NC stage > 1. The UDS parameters utilized in this chart were the maximum values experienced at any point in the research volunteers’ longitudinal course on study. In addition to those BPSD subtypes, final CDR assessments were used for language dysfunction and global cognitive impairment. The severity of multiple BPSD subtypes trended to be worse in more advanced Braak NFT stages. Asterisks indicate statistical significance: *(*p* < 0.05), **(*p* < 0.01), ***(*p* < 0.001): these are nominal *p* values, using Chi-square test with 5 degrees of freedom

**Fig. 3:**
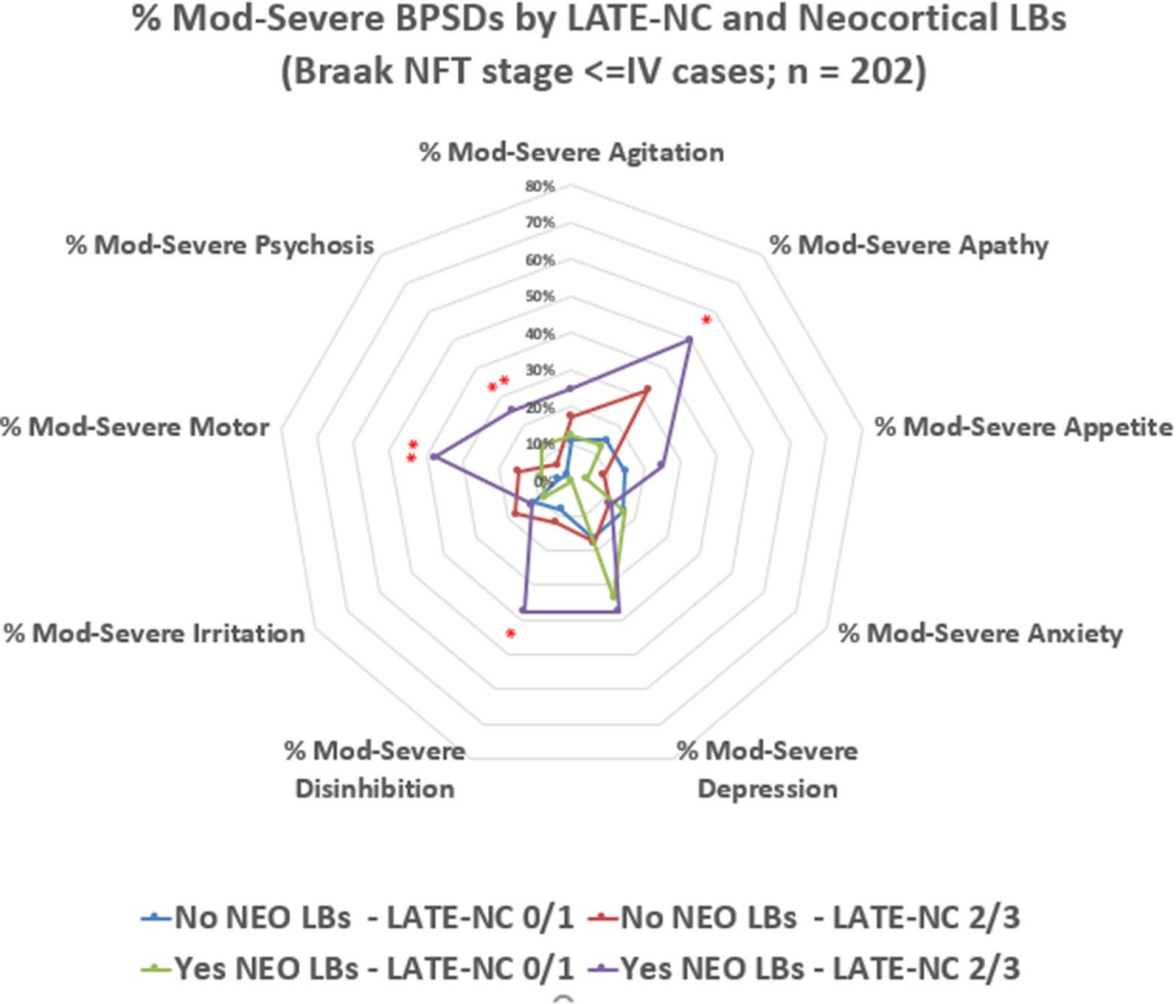
Radar chart depicts the percent of cases with moderate or severe BPSD subtypes, stratified by presence or absence of neocortical LBs and LATE-NC Stage > 1, among cases lacking severe ADNC (i.e., Braak NFT stages < V). Cases included for this chart were selected among participants (n = 202) that lacked severe ADNC. A number of the BPSD subtypes were more severe on average in cases with both LATE-NC and neocortical LBs. Asterisks indicate statistical significance: *(*p* < 0.05), **(*p* < 0.01), ***(*p* < 0.001): these are nominal *p* values, using Chi-square test with 3 degrees of freedom

**Fig. 4:**
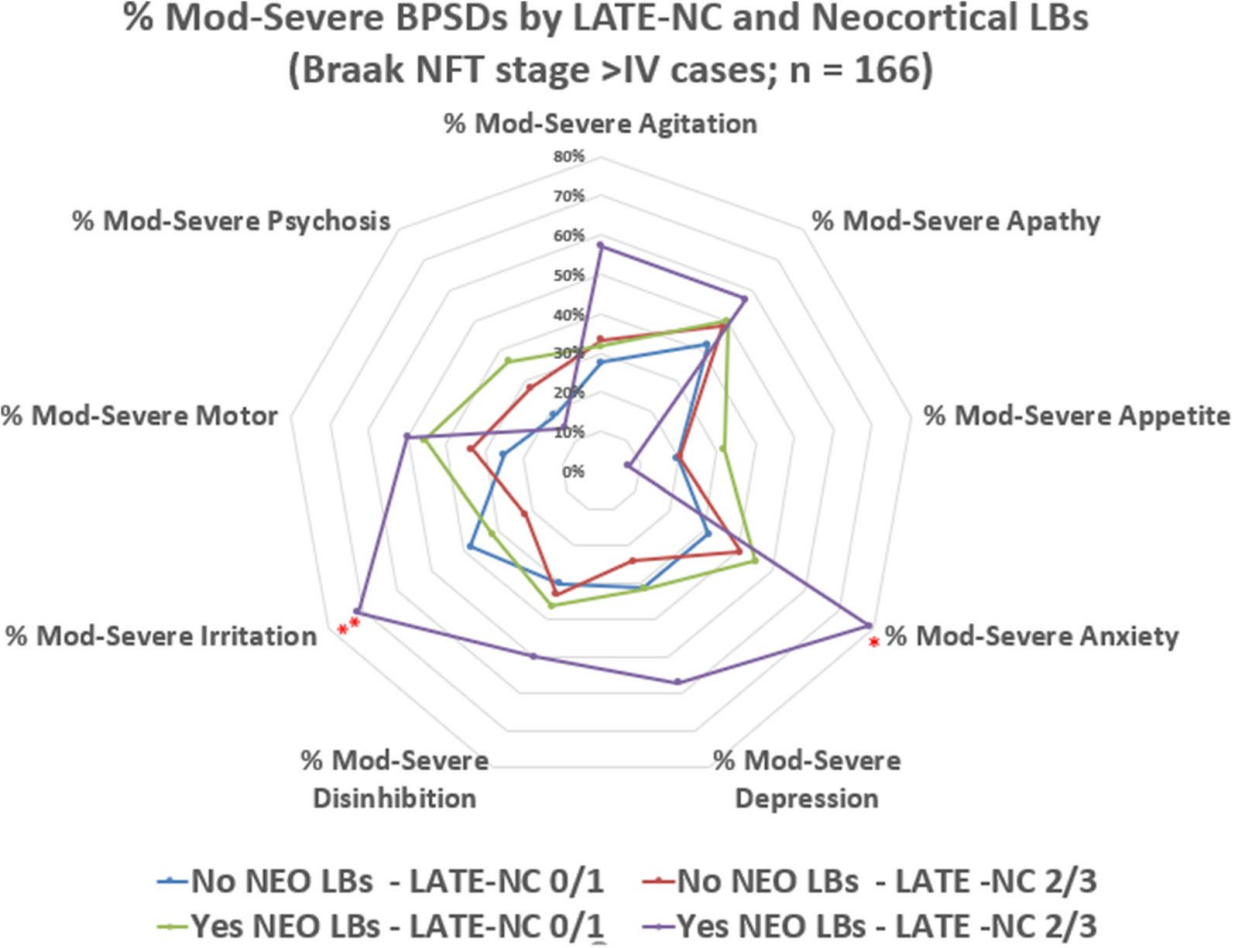
Radar chart depicts the percent of cases with moderate or severe BPSD subtypes, stratified by presence or absence of neocortical LBs and LATE-NC Stage > 1, among cases with severe ADNC (i.e., Braak NFT stages V or VI). Cases included for this chart were selected among participants with severe ADNC (n = 166). As was true in cases lacking severe ADNC, some of the BPSD subtypes were more severe on average in subjects with both LATE-NC stage > 1 and neocortical LBs. Asterisks indicate statistical significance: *(*p* < 0.05), **(*p* < 0.01), ***(*p* < 0.001): these are nominal *p* values, using Chi-square test with 3 degrees of freedom

Radar charts were used to summarize the distribution of severe BPSD across pathology groups (Figs. 2, 3, 4). In cases lacking comorbid neocortical LBs or LATE-NC stage > 1, there was an increase in the number of BPSDs when comparing between Braak NFT stage VI and Braak NFT stage V (Fig. 2). This trend applied to multiple BPSD subtypes. Those with more co-pathologies tended to have severe clinical phenotypes, including more BPSD subtypes. The importance of comorbid neocortical LBs and also LATE-NC stage > 1 neuropathologies could be observed both in the presence and absence of severe ADNC, as shown in Figs. 3 and 4. *p* values for each chi-square are reported in Additional file 2: Table S6.

Tables 7 and 8 present the results in a different format, testing the results for combinations of pathologies when comparing groups with BPSD subtype ratings of 2 or 3 versus 0 or 1. Data on “pure” non-ADNC pathologies (lacking additional strong co-pathologies) are shown in Table 7. LATE-NC stage > 1 was associated with global cognitive impairment (*p* = 0.004), motor disturbance (*p* = 0.008), and apathy (*p* = 0.02). Pure neocortical LBs was associated with depression (*p* = 0.04). By contrast, ADNC (Braak NFT stages > IV) (Table 8) was associated with more numerous and severe BPSD subtypes.

**Table 7:** Odds ratio and 95% confidence intervals (OR and 95% CI) of having moderate or severe BPSD (scored 2 or 3 on 0–3 scale), stratified by pathology, in cases with Braak NFT stage < V and relatively pure subtypes of pathology

Symptom readout	LATE-NC only versus control		Neocortical Lewy bodies only versus control		Vascular pathology^a^ only versus control	
	OR (95% CI)	*p* value^b^	OR (95% CI)	*p* value^b^	OR (95% CI)	*p* value^b^
CDR-Global	4.3 (1.6–11.9)	**0.004**	1.0 (0.2–4.3)	0.96	11.9 (2.7–86)	**0.003**
CDR-Language	1.5 (0.2–7.3)	0.64	*N/A*^d^	*N/A*^d^	9.7 (1.4–199)	**0.048**
NPI-Q: Psychosis^c^	2.6 (0.7–8.7)	0.14	2.9 (0.6–12.5)	0.16	3.1 (0.7–14.3)	0.13
NPI-Q: Agitation	1.6 (0.5–4.6)	0.40	1.2 (0.3–4.1)	0.79	1.7 (0.5–5.1)	0.36
NPI-Q: Depression	1.1 (0.4–2.9)	0.84	2.8 (1.0–7.4)	**0.041**	1.2 (0.4–3.3)	0.69
NPI-Q: Anxiety	0.9 (0.2–2.8)	0.89	1.2 (0.3–3.7)	0.77	1.4 (0.5–4.0)	0.48
NPI-Q: Apathy	2.9 (1.2–7.1)	**0.021**	0.8 (0.2–2.6)	0.71	1.7 (0.6–4.6)	0.30
NPI-Q: Disinhibition	2.0 (0.5–6.8)	0.30	*N/A*^d^	*N/A*^d^	3.9 (1.1–16.4)	**0.043**
NPI-Q: Irritability	2.0 (0.6–5.7)	0.21	0.7 (0.1–2.9)	0.66	2.3 (0.8–7.1)	0.14
NPI-Q: Motor	7.0 (1.7–31.6)	**0.008**	2.7 (0.4–14.5)	0.26	3.5 (0.5–29.4)	0.20
NPI-Q: Appetite	0.5 (0.1–1.8)	0.36	0.2 (0.0–1.2)	0.17	1.3 (0.5–3.5)	0.63

^a^“Vascular pathology” describes cases where it was deemed likely that cerebrovascular disease was severe enough to contribute to cognitive impairment

^b^*p* < 0.05 in bold

^c^“Psychosis” combines the NPI-Q fields of delusions and hallucinations

^d^In several categories (indicated by “N/A”), the sample sizes were too small to perform valid statistical tests

**Table 8:** Odds ratio and 95% confidence intervals (OR and 95% CI) of having moderate or severe BPSD (scored 2/3 versus 0/1, on 0–3 scale), stratified by pathology, in cases with severe ADNC (Braak NFT stages V or VI)

Symptom readout	Severe ADNC only versus control		Severe ADNC + LATE-NC Stg > 1 versus control		Severe ADNC + neocortical LBs versus control		QMP^a^ versus control	
	OR (95% CI)	*p* value^b^	OR (95% CI)	*p* value^b^	OR (95% CI)	*p* value^b^	OR (95% CI)	*p* value^b^
CDR-Global	10.3 (4.8–23.8)	** < 10**^**−6**^	18.1 (7.8–46.3)	** < 10**^**−6**^	44.4 (12.6–202.2)	** < 10**^**−6**^	37.2 (9.2–204.7)	**0.000003**
CDR-Language	7.0 (2.6–21.0)	**0.002**	7.1 (2.5–22.8)	**0.0005**	21.7 (5.8–95.2)	**0.00001**	21.4 (5.0–109.0)	**0.00007**
NPI-Q: Psychosis^c^	7.0 (2.3–26.2)	**0.015**	18.0 (5.8–71.1)	**0.000004**	21.3 (5.5–98.0)	**0.00002**	50.6 (11.3–294.2)	**0.000001**
NPI-Q: Agitation	3.4 (1.5–7.5)	**0.027**	4.2 (1.9–9.8)	**0.0005**	3.5 (1.1–11.1)	**0.035**	13.3 (3.6–55.1)	**0.0001**
NPI-Q: Depression	2.4 (1.2–5.0)	**0.013**	1.7 (0.8–3.9)	0.18	2.6 (0.8–8.0)	0.091	7.4 (2.2–26.8)	**0.002**
NPI-Q: Anxiety	2.0 (1.0–4.1)	**0.049**	3.9 (1.8–8.7)	**0.0005**	2.8 (1.0–8.1)	0.051	15.8 (4.3–76.7)	**0.0001**
NPI-Q: Apathy	4.2 (2.1–8.7)	**0.00008**	6.5 (3.0–14.5)	**0.000002**	5.0 (1.7–14.9)	**0.003**	10.0 (2.9–38.9)	**0.0004**
NPI-Q: Disinhibition	5.0 (2.2–12.2)	**0.0002**	6.6 (2.0–16.9)	**0.00004**	4.7 (1.4–15.6)	**0.01**	13.0 (3.5–53.3)	**0.0002**
NPI-Q: Irritability	4.3 (2.1–9.3)	**0.0001**	2.1 (0.8–5.0)	0.11	2.6 (0.8–8.0)	0.11	18.2 (5.1–79.1)	**0.00002**
NPI-Q: Motor	6.1 (2.1–20.0)	**0.001**	17.2 (5.8–61.8)	**0.000002**	14.2 (3.8–59.7)	**0.0001**	23.1 (5.5–112.1)	**0.00003**
NPI-Q: Appetite	1.3 (0.6–2.8)	0.52	1.4 (0.6–3.3)	0.40	3.5 (1.1–11.3)	**0.032**	0.4 (0–2.4)	0.42

^a^QMP indicates the quadruple misfolded protein phenotype: Braak NFT stages V or VI, LATE-NC stage > 1, and neocortical LBs

^b^*p* < 0.05 in bold

^c^“Psychosis” combines the NPI-Q fields of delusions and hallucinations

ADNC = Severe Alzheimer’s disease neuropathological changes; Braak NFT stages V or VI; LATE-NC = limbic predominant age-related TDP-43 encephalopathy neuropathologic changes; LBs = Lewy bodies

Secondary clinical-pathologic association tests were performed, focusing on additional comparisons of BPSDs between subsets of included individuals. These tests also served as sensitivity analyses. We queried the association between BPSDs in LATE-NC cases with HS versus without HS, to evaluate the associative impact of HS (Additional file 3: Fig. S1); compared PART (CERAD neuritic plaque levels of “none”) with Braak NFT stages 0-II versus stages III/IV (Additional file 3: Fig. S2); and also tested if there were differences in BPSDs in ADNC cases with versus without amygdala-only Lewy bodies (Additional file 3: Fig. S3). We also assessed separately the subset of cases that lacked moderate or severe dementia. In this analysis of cases with global CDR scores = 0, 0.5, or 1 we assessed the correlative impact of LATE-NC, severe ADNC, and neocortical LBs (Additional file 3: Figs. S4–S6). Summary data for these secondary analyses are presented in Table 9. Collectively, these results again underscored the particularly strong associations between tau/NFT pathology and BPSDs.

**Table 9:** Secondary analyses to understand correlations between BPSDs with hippocampal sclerosis (HS), primary age-related tauopathy (PART), amygdala Lewy bodies, and the observations among CDR 0–1 subjects

Comparison Group	n	Control Group	n	Comparison group with more BPSD than control group (*p* value)	Suppl. figures
LATE-NC < = 1 + Yes HS	24	LATE-NC* < = 1 + No HS	230	None	Additional file 3: Fig. S1
LATE-NC > 1 + Yes HS	90	LATE-NC* > 1 + No HS	20	None	Additional file 3: Fig. S1
PART Braak NFT stage III/IV	15	PART* Braak NFT stage 0-II	75	Irritation (*p* = 0.008) Agitation (*p* = 0.006) Psychosis (*p* = 0.001)	Additional file 3: Fig. S2
ADNC w/ amygdala LBs (No NEO LBs)	22	ADNC* w/o amygdala LBs (No NEO LBs)	108	None	Additional file 3: Fig. S3
CDR 0/0.5/1 YES LATE-NC	53	CDR 0/0.5/1 NO LATE-NC*	190	None	Additional file 3: Fig. S4
CDR 0/0.5/1 YES ADNC	69	CDR 0/0.5/1 NO ADNC*	174	Psychosis (*p* < 0.0001) Agitation (*p* = 0.004) Anxiety (*p* = 0.02) Apathy (*p* = 0.003) Motor (*p* = 0.03) Disinhibition (*p* = 0.0001) Irritation (*p* = 0.004) Appetite (*p* = 0.03)	Additional file 3: Fig. S5
CDR 0/0.5/1 YES Neocortical LBs	34	CDR 0/0.5/1 NO Neocortical LBs	209	None	Additional file 3: Fig. S6

*ADNC indicates Braak NFT stage > IV; PART indicates CERAD neuritic plaque score of “none”; LATE-NC indicates LATE-NC stage > 1

Two BPSD subtypes that may be associated with frontal lobe dysfunction are disinhibition and language disturbances [56, 78]. LATE-NC has also been shown to affect frontal and temporal brain regions [83]. Sensitivity analyses were run comparing LATE-NC stage 0 versus stages 1/2/3 and these results are shown in Additional file 2: Tables S4 and S5. Inclusion of mild disinhibition in the clinical operationalization showed slightly different results including an association between LATE-NC with disinhibition (*p* < 0.01). Our analyses also provided clues about whether individuals with severe disinhibition or language disturbance were particularly likely to have pure or mixed LATE-NC patterns. As shown in Tables 10 and 11, there was a relatively high frequency of LATE-NC among persons with severe disinhibition or language problems (~ 50% and ~ 40%, respectively). However, the frequency of severe ADNC (Braak stages > IV) was even higher in association with these clinical phenotypes (~ 80% and ~ 90%, respectively). Neither disinhibition nor language dysfunction was a reliable indicator of LATE-NC—those BPSD subtypes were more likely to signal the presence of severe ADNC (*p* < 0.001), and there also were trends for associations between vascular pathologies and both disinhibition and language disorder (*p* < 0.05; Table 7).

**Table 10:** Disinhibition (by UDS assessment instrument): numbers, % LATE-NC, % ADNC

Disinhibition severity	N	% LATE-NC (Stage > 1)*	% ADNC (Braak NFT stage > IV)*
0	235	21.7	31.5
1	59	45.8	61.0
2	41	36.6	73.2
3	33	51.5	78.8

*UDS* Uniform data set, *LATE-NC* Limbic predominant age-related TDP-43 encephalopathy neuropathologic changes, *ADNC* Alzheimer’s disease neuropathological changes, *NFT* Neurofibrillary tangle

*Includes all cases with the pathology (not just neuropathologically “pure” cases)

**Table 11:** Language disturbance (by CDRLANG assessment instrument): numbers, % LATE-NC, % ADNC

Language problem severity	N	% LATE-NC (stage > 1)*	% ADNC (Braak NFT stage > IV)*
0	185	21.6	28.1
0.5	57	42.1	52.6
1	28	35.7	67.9
2	25	40.0	80.0
3	37	37.8	89.2

*Includes all cases with the pathology (not just neuropathologically “pure” cases)

*CDR* Clinical dementia rating, *LATE-NC* Limbic predominant age-related TDP-43 encephalopathy neuropathologic changes, *ADNC* Alzheimer’s disease neuropathological changes, *NFT* neurofibrillary tangle

## Discussion

**Fig. 5:**
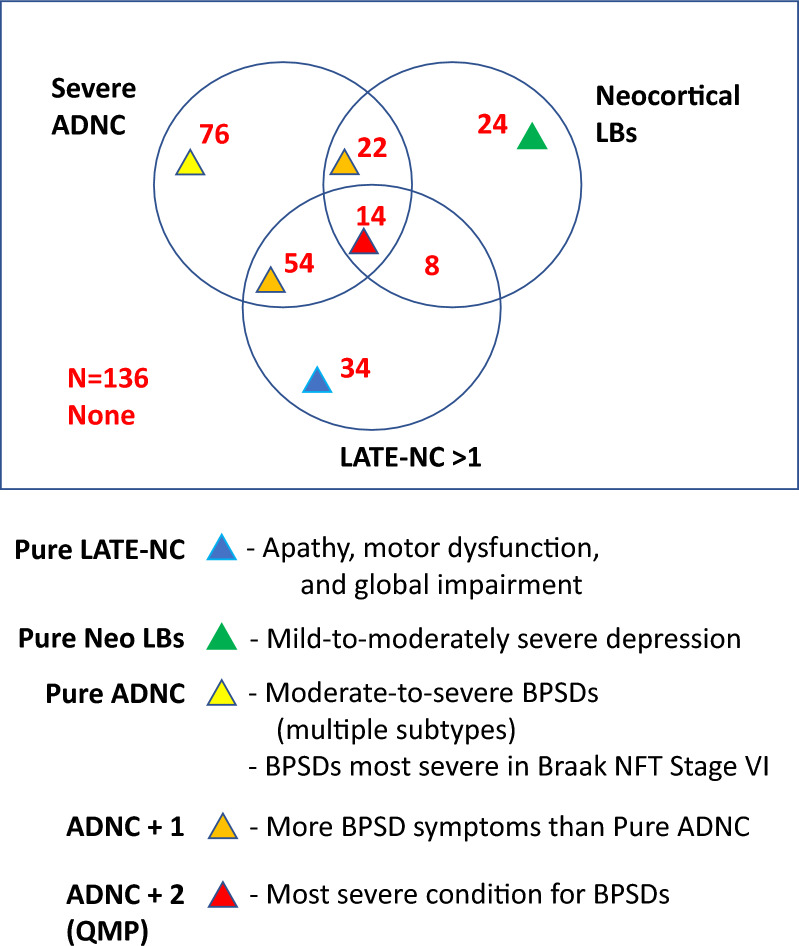
Non-proportional Venn diagram depicts the numbers of included cases in the present study according to various pathologic combinations, along with a summary list of the main BPSD subtypes associated with those pathology-defined categories

The present study analyzed the relationships between clinically-documented BPSD subtypes and various neuropathologies in the UK-ADRC autopsy cohort. Our findings confirm that in aged brains, both elements of clinical-pathological correlations are complex—the clinical manifestations of brain diseases are heterogeneous and often combinatorial, as are the underlying pathologies. The results of the present study are illustrated in schematic form in Fig. 5, with a representation of the distribution of cases stratified by neuropathological findings, and the BPSD subtypes that were associated with those neuropathologies.

Prior studies have helped characterize neurodegenerative diseases and their clinical manifestations, which included BPSD subtypes. Some prior studies lacked autopsy results (diseases were defined according to clinical criteria or biomarker/neuroimaging findings), whereas in many other prior studies, the diversity of both BPSD and neuropathological findings were not fully represented. Perhaps due to those variations in study designs, there was some divergence in the prior studies’ findings with regard to the underlying hypothesized pathologic substrates of BPSD (see for example refs [6, 12, 25, 28, 29, 31, 32, 35, 36, 41, 43, 61, 95, 104, 107, 108, 111, 120, 121, 124, 125]). According to a prior review of studies focusing on psychosis in clinically diagnosed AD, the incidence of delusions and hallucinations increases over three years post diagnosis, while greater impairment in global cognition is also associated with higher prevalence of psychosis [99]. Notable aspects of the clinical-pathological correlations (with a panel of BPSDs) in the present study included assessments of LATE-NC, PART, amygdala LBs in ADNC, HS, and various combinations of mixed pathologies.

A subset of relevant published studies analyzed data from the NACC Neuropathology Data Set [9]. These studies used the UDS [8] with correlation among multiple different AD research centers [9]. In a series of articles by David Munoz and colleagues, BPSD subtypes including psychosis, agitation, and others were associated with both neurodegenerative and vascular pathologies with a lesser correlative emphasis on ADNC severity [36, 57, 96, 105]. By contrast, Malpas et al. found that Braak NFT staging was associated with neuropsychiatric symptoms [65] and Katsumata et al. reported that QMP (often with severe ADNC) was associated with delusions, hallucinations, and other BPSD [54]. Additional insights were obtained via analyses of the NACC data set about the associations between LBP and BPSD [19, 94, 95], and/or LATE-NC and BPSD [10, 40, 70, 105] (see below). Genetic tests using the NACC neuropathology data set and other information have indicated that there may be genetic risk factors for “mixed pathology” combinations [26].

Our findings were generally in agreement with prior work. Strengths of the current study included textured documentation of clinical and pathological features. Most research volunteers were recruited into the longitudinal study with normal cognitive (and other neurological) status, and then were followed for many years. BPSD assessments were not dichotomous but rather their severity was scored on a 0–3 scale. Likewise, the pathological features were graded using semi-quantitative neuropathologic staging metrics. Perhaps due to these strengths, and despite drawbacks of studying a single cohort with a limited range of ethnoracial diversity (see below), some patterns emerged from our analyses.

A conspicuous finding of our study was that ADNC (as operationalized with Braak NFT stages) had a large correlative impact on BPSD. Even in cases lacking substantial neocortical LBP or LATE-NC, the presence of severe ADNC was often associated with multiple BPSD subtypes. Our results also underscore that the contradistinction between Braak NFT stages V and VI (both commonly grouped together to indicate “severe ADNC”) is important—there were substantial differences between the correlative impact of pure Braak NFT stages V and VI in terms of BPSD. This indicates that widespread neocortical tauopathy is a driver of BSPD, consistent with prior work [65]. However, as reported in prior studies [13, 52], pure ADNC only represented < 25% of subjects, and, pathologic stage for stage, pure ADNC also was associated with fewer BPSD subtypes, in comparison to the cases with comorbid pathologies (LBP and LATE-NC). For a given patient with multiple (often > 10) different BPSD subtypes, QMP was often the underlying pathologic substrate.

Unlike ADNC, LBP was associated more strongly with BPSD than with global cognition, yet this trend generally was most notable in cases with comorbid ADNC. The neuropathologic phenotype of ADNC + LBP had particular associations with neuropsychiatric symptoms such as psychoses and depression. More severe BPSDs were seen with ADNC + LBP + LATE-NC (see below), except this was not the case with psychoses; rather, the trend was for the pathologies of ADNC + LBP to be associated with the psychoses (Fig. 4). Some of these results may be epiphenomena related to many variables being assessed with a limited sample size. However, overall these findings are compatible with prior studies – LBP was associated with neuropsychiatric disease previously, and specific relevant nuclei of the cerebrum and brainstem are vulnerable to LBP [16, 17, 44, 95, 96, 117]. Prior work also has emphasized that LBP should not be viewed in isolation because the ADNC severity plays an important role in modifying the clinical phenotype. For example, Gibson et al. [43] and Pillai et al. [95] both reported that people with the combined ADNC + LBP phenotype had the highest risk of BPSD including hallucinations, agitation, and apathy. The data from our study are compatible with those prior results.

One issue that has generated divergent perspectives is the question of whether (and to what degree) LATE-NC is associated with BPSD. LATE-NC stage > 1 has been consistently associated with episodic memory loss and global cognitive impairment, independent of ADNC and other co-pathologies [77, 81, 83]. Liu et al. reported that in persons with comorbid ADNC, LATE-NC was not associated with additive neuropsychiatric symptoms [64]. By contrast, Munoz et al. described that age-related TDP-43 pathology was associated with agitation or aggression [105]. Gauthreaux et al. reported that among individuals with low or intermediate ADNC severities, those with comorbid LATE-NC had a higher prevalence of apathy, disinhibition, agitation, and personality change [39]. In that well-powered study, differences in comparing LATE-NC versus no LATE-NC cases were less evident in the group with severe ADNC [39]. There also is an open question as to how the symptomatology of LATE-NC is correlated with FTLD. FTLD is a term that was coined to describe pathologies that underlie the FTD clinical syndrome (with frontal lobe dysfunction) [58, 78], i.e. behavioral disinhibition and/or language problems. Jung et al. [51] and Teylan et al. [115] found considerable differences between FTLD-TDP and LATE-NC in terms of clinical manifestations; these clinical distinctions are also mirrored by pathological differences between FTLD-TDP and LATE-NC [98]. In the present study, we found a modest increase in disinhibition in LATE-NC (see Additional file 2: in Table S4, all cases with any disinhibition including mild cases were compared with “non”) and a marginal trend for language dysfunction in LATE-NC versus low-pathology controls. However, both disinhibition and language problems were not specific since ADNC and vascular pathologies were more strongly associated with those BPSD than LATE-NC was. If cases with disinhibition were disproportionately present in any subset of cases according to neuropathology, it would be the QMP group.

The present study had limitations. The UK-ADRC research volunteers are highly educated, and most autopsied volunteers were White [102]; these sampling characteristics limit generalizability to other populations. There also was a bias toward risk for AD-type pathology, including *APOE* ε4 allele rate of almost 39% (population prevalence is ~ 25% [21, 23]). This bias is associated with increased ADNC and a corresponding decrease of “pure” LATE-NC and/or LBP subtypes. It is imperative that future studies incorporate more diverse participants—in both ethnoracial and socioeconomic terms [5]. Potential confounders were not assessed, including medications that may either treat or exacerbate BPSD, and we did not factor in many other potential comorbid conditions. Any given BPSD subtype (for example, depression) could merit its own separate study and additional careful subtyping. There also are many possible ways to operationalize each of the pathologic variables, including both the neurodegenerative (*e.g*., misfolded Aβ, Tau, α-Synuclein, and TDP-43 proteinopathies) and cerebrovascular pathologies (large infarcts, small infarcts, arteriolosclerosis, CAA, etc.). In the future we may be able to analyze larger numbers of cases and generate new statistical tools to assess the various parameters (and their combinations) comparatively.

## Conclusions

We studied the clinical-pathological associations related to BPSD subtypes in the UK-ADRC autopsy cohort. This study was novel in that it included a range of pathologies including ADNC, LBP, LATE-NC, PART, HS, and pathologic combinations. We also studied a broad range of BPSDs, rather than only mood disorders and psychoses. In this community-based sample, most demented subjects had mixed pathologies, and BPSD subtypes tended to be more numerous and more severe with Braak NFT stage VI ADNC (versus Braak NFT stage V or below), with even more severe BPSDs in cases with comorbid LATE-NC and/or LBP. LATE-NC alone was not strongly associated with FTLD-like BPSDs, relative to ADNC alone. Despite there being intriguing correlations between BPSD subtypes and pathologic patterns, the presence and severity of a given BPSD were not reliably associated specifically with any pathologic subtype or combination of subtypes.

## Supplementary Information

**Additional file 1: Table S1.** Operationalization of BPSD subtypes.

**Additional file 2: Table S2.** Average raw numbers of different BPSD subtypes per individual participant by pathology category. **Table S3.** Sample sizes of subsets of cases, stratified by pathological features. **Table S4.** Odds ratio (and 95% CI) of having any degree of BPSD (scored 1, 2, or 3 versus none), stratified by pathology, in cases with Braak NFT stage < V and relatively pure subtypes of pathology; analogous to Table 5. **Table S5.** Odds ratio (and 95% CI) of having any degree of BPSD (scored 1, 2, or 3 versus none), stratified by pathology, in cases with Braak NFT stages V or VI; analogous to Table 6. **Table S6.** *p* values for radar charts (Figs. 2, 3, 4), stratified by BPSD subtypes.

**Additional file 3: Figure S1.** Radar chart depicts the percent of cases with moderate or severe BPSD subtypes, stratified by presence or absence of hippocampal sclerosis and LATE-NC Stage > 1. Asterisks indicate statistical significance: *(*p* < 0.05), **(*p* < 0.01), ***(*p* < 0.001): these are nominal *p* values. Statistical Tests: 2 sets of Chi-squares: (1) looking at No/Yes HS within LATE <1 and (2) looking at No/Yes HS within LATE > 1. Within both sets of analyses none of the BPSDs had a *p* val <0.05. For summary information, see Table 9. **Figure S2.** Radar chart depicts the percent of cases with moderate or severe BPSD subtypes, stratified by severity of PART (i.e., all cases have CERAD neuritic amyloid plaque scores of “none” and we compared Braak NFT stages 0-II vs III/IV). Asterisks indicate statistical significance: *(*p* < 0.05), **(*p* < 0.01), ***(*p* < 0.001): these are nominal *p* values, using Chi-square test. For summary information, see Table 9. **Figure S3.** Radar chart depicts the percent of cases with moderate or severe BPSD subtypes, stratified by presence or absence amygdala Lewy bodies (LBs), among cases with severe ADNC (i.e., Braak NFT stages V or VI). Asterisks indicate statistical significance: *(*p* < 0.05), **(*p* < 0.01), ***(*p* < 0.001): these are nominal *p* values, using Chi-square test. For summary information, see Table 9. **Figure S4.** Radar chart depicts the percent of cases with moderate or severe BPSD subtypes, stratified by presence or absence of LATE-NC Stage > 1, among cases lacking moderate or severe dementia (i.e., CDR global scores = 0, 0.5, or 1). For summary information, see Table 9. **Figure S5.** Radar chart depicts the percent of cases with moderate or severe BPSD subtypes, stratified by presence or absence of severe ADNC (Braak NFT stages > IV), among cases lacking moderate or severe dementia (i.e., CDR global scores = 0, 0.5, or 1). Asterisks indicate statistical significance: *(*p* < 0.05), **(*p* < 0.01), ***(*p* < 0.001): these are nominal *p* values, using Chi-square test. For summary information, see Table 9. **Figure S6.** Radar chart depicts the percent of cases with moderate or severe BPSD subtypes, stratified by presence or absence of neocortical Lewy bodies (LBs), among cases lacking moderate or severe dementia (i.e., CDR global scores = 0, 0.5, or 1). For summary information, see Table 9.

## Data Availability

The datasets used and/or analysed during the current study will be made available from the corresponding author (PTN) on reasonable request.

## Abbreviations

ADNC: Alzheimer’s disease neuropathologic changes
BPSD: Behavioral and psychological symptoms of dementia
CAA: Cerebral amyloid angiopathy
FTD: Frontotemporal dementia
FTLD-TDP: Frontotemporal lobar degeneration with TDP-43 inclusions
HS: Hippocampal sclerosis
LATE-NC: Limbic predominant age-related TDP-43 encephalopathy neuropathologic changes
LBD: Lewy body disease
NACC: National Alzheimer’s Disease Coordinating Center
NFT: Neurofibrillary tangle
NPI-Q: Neuropsychiatric Inventory Questionnaire
PART: Primary age-related tauopathy
QMP: Quadruple misfolded proteinopathy
UDS: Uniform Data Set
UK-ADRC: University of Kentucky Alzheimer’s Disease Research Center
