# Supplementary material for: Neurodegenerative pathologies associated with behavioral and psychological symptoms of dementia in a community-based autopsy cohort
Source: Acta Neuropathol Commun. 2023 Jun 2;11:89. doi: 10.1186/s40478-023-01576-z (PMC10236713; doi:10.1186/s40478-023-01576-z)
Supplement: Supplementary file 1 — Additional file 1: Table S1. Operationalization of BPSD subtypes. [file 40478_2023_1576_MOESM1_ESM.docx]

**Supplemental Table 1.** Operationalization of BPSD subtypes

| BPSD subtype | | Source of assessment instrument | Severity scale | Wording of assessment instrument | |
| --- | --- | --- | --- | --- | --- |
| Delusions | | Form B5: Behavioral assessment -- Neuropsychiatric Inventory Questionnaire (NPI-Q)* | 0-3 | Does the patient believe that others are stealing from him or her, or planning to harm him or her in some way? (0-3 scale denotes severity of symptoms) | |
| Hallucinations | | Form B5: Behavioral assessment -- Neuropsychiatric Inventory Questionnaire (NPI-Q)* | 0-3 | Does the patient act as if he or she hears voices? Does he or she talk to people who are not there? (0-3 scale denotes severity of symptoms) | |
| Agitation or aggression | | Form B5: Behavioral assessment -- Neuropsychiatric Inventory Questionnaire (NPI-Q)* | 0-3 | Is the patient stubborn and resistive to help from others? (0-3 scale denotes severity of symptoms) | |
| Depression or dysphoria | | Form B5: Behavioral assessment -- Neuropsychiatric Inventory Questionnaire (NPI-Q)* | 0-3 | Does the patient act as if he or she is sad or in low spirits? Does he or she cry? (0-3 scale denotes severity of symptoms) | |
| Anxiety | | Form B5: Behavioral assessment -- Neuropsychiatric Inventory Questionnaire (NPI-Q)* | 0-3 | Does the patient become upset when separated from you? Does he or she have any other signs of nervousness, such as shortness of breath, sighing, being unable to relax, or feeling excessively tense? (0-3 scale denotes severity of symptoms) | |
| Apathy or indifference | | Form B5: Behavioral assessment -- Neuropsychiatric Inventory Questionnaire (NPI-Q) | 0-3 | Does the patient seem less interested in his or her usual activities and in the activities and plans of others? (0-3 scale denotes severity of symptoms) | |
| Disinhibition | | Form B5: Behavioral assessment -- Neuropsychiatric Inventory Questionnaire (NPI-Q) | 0-3 | Does the patient seem to act impulsively? For example, does the patient talk to strangers as if he or she knows them, or does the patient say things that may hurt people’s feelings? (0-3 scale denotes severity of symptoms) | |
| Irritability or lability | | Form B5: Behavioral assessment -- Neuropsychiatric Inventory Questionnaire (NPI-Q) | 0-3 | Is the patient impatient or cranky? Does he or she have difficulty coping with delays or waiting for planned activities? (0-3 scale denotes severity of symptoms) | |
| Appetite or eating problem | | Form B5: Behavioral assessment -- Neuropsychiatric Inventory Questionnaire (NPI-Q)* | 0-3 | Has the patient lost or gained weight, or had a change in the food he or she likes? (0-3 scale denotes severity of symptoms) | |
| Motor disturbance | | Form B5: Behavioral assessment -- Neuropsychiatric Inventory Questionnaire (NPI-Q)* | 0-3 | Does the patient engage in repetitive activities, such as pacing around the house, handling  buttons, wrapping string, or doing other things repeatedly? (0-3 scale denotes severity of symptoms) | |
| Language status/disturbance | | Supplemental CDR** | 0-3 | 0: No language difficulty; 0.5: Consistent mild word finding difficulties; 1: Moderate word finding difficulty; 2: Moderate to severe impairment; 3: Severe deficit; no intelligible speech. | |
| Global cognitive status/impairment | | CDR Dementia Staging Calculator*** | 0-3 | 0-3 score encompasses domains of Memory, Orientation, Judgment/Problem-solving, Community affairs, Home and hobbies, and Personal Care to convey impact of disease on activities of daily living | |
| **https://files.alz.washington.edu/documentation/uds1-ivp-guidebook.pdf; | | | |  |  |
| ***https://sites.cscc.unc.edu/aric/sites/default/files/public/forms/CDS%20Clinical%20Dementia%20Rating%20-%20Summary%20and%20QxQ.pdf | | | |  |  |
| ****https://naccdata.org/data-collection/tools-calculators/cdr | | | |  |  |
