# Supplementary material for: Neurodegenerative pathologies associated with behavioral and psychological symptoms of dementia in a community-based autopsy cohort
Source: Acta Neuropathol Commun. 2023 Jun 2;11:89. doi: 10.1186/s40478-023-01576-z (PMC10236713; doi:10.1186/s40478-023-01576-z)
Supplement: Supplementary file 3 — Additional file 3: Figure S1. Radar chart depicts the percent of cases with moderate or severe BPSD subtypes, stratified by presence or absence of hippocampal sclerosis and LATE-NC Stage > 1. Asterisks indicate statistical significance: *(p < 0.05), **(p < 0.01), ***(p < 0.001): these are nominal p values. Statistical Tests: 2 sets of Chi-squares: (1) looking at No/Yes HS within LATE <1 and (2) looking at No/Yes HS within LATE > 1. Within both sets of analyses none of the BPSDs had a p val <0.05. For summary information, see Table 9. Figure S2. Radar chart depicts the percent of cases with moderate or severe BPSD subtypes, stratified by severity of PART (i.e., all cases have CERAD neuritic amyloid plaque scores of “none” and we compared Braak NFT stages 0-II vs III/IV). Asterisks indicate statistical significance: *(p < 0.05), **(p < 0.01), ***(p < 0.001): these are nominal p values, using Chi-square test. For summary information, see Table 9. Figure S3. Radar chart depicts the percent of cases with moderate or severe BPSD subtypes, stratified by presence or absence amygdala Lewy bodies (LBs), among cases with severe ADNC (i.e., Braak NFT stages V or VI). Asterisks indicate statistical significance: *(p < 0.05), **(p < 0.01), ***(p < 0.001): these are nominal p values, using Chi-square test. For summary information, see Table 9. Figure S4. Radar chart depicts the percent of cases with moderate or severe BPSD subtypes, stratified by presence or absence of LATE-NC Stage > 1, among cases lacking moderate or severe dementia (i.e., CDR global scores = 0, 0.5, or 1). For summary information, see Table 9. Figure S5. Radar chart depicts the percent of cases with moderate or severe BPSD subtypes, stratified by presence or absence of severe ADNC (Braak NFT stages > IV), among cases lacking moderate or severe dementia (i.e., CDR global scores = 0, 0.5, or 1). Asterisks indicate statistical significance: *(p < 0.05), **(p < 0.01), ***(p < 0.001): these are nominal p values, usi [file 40478_2023_1576_MOESM3_ESM.pptx]

## Slide 1
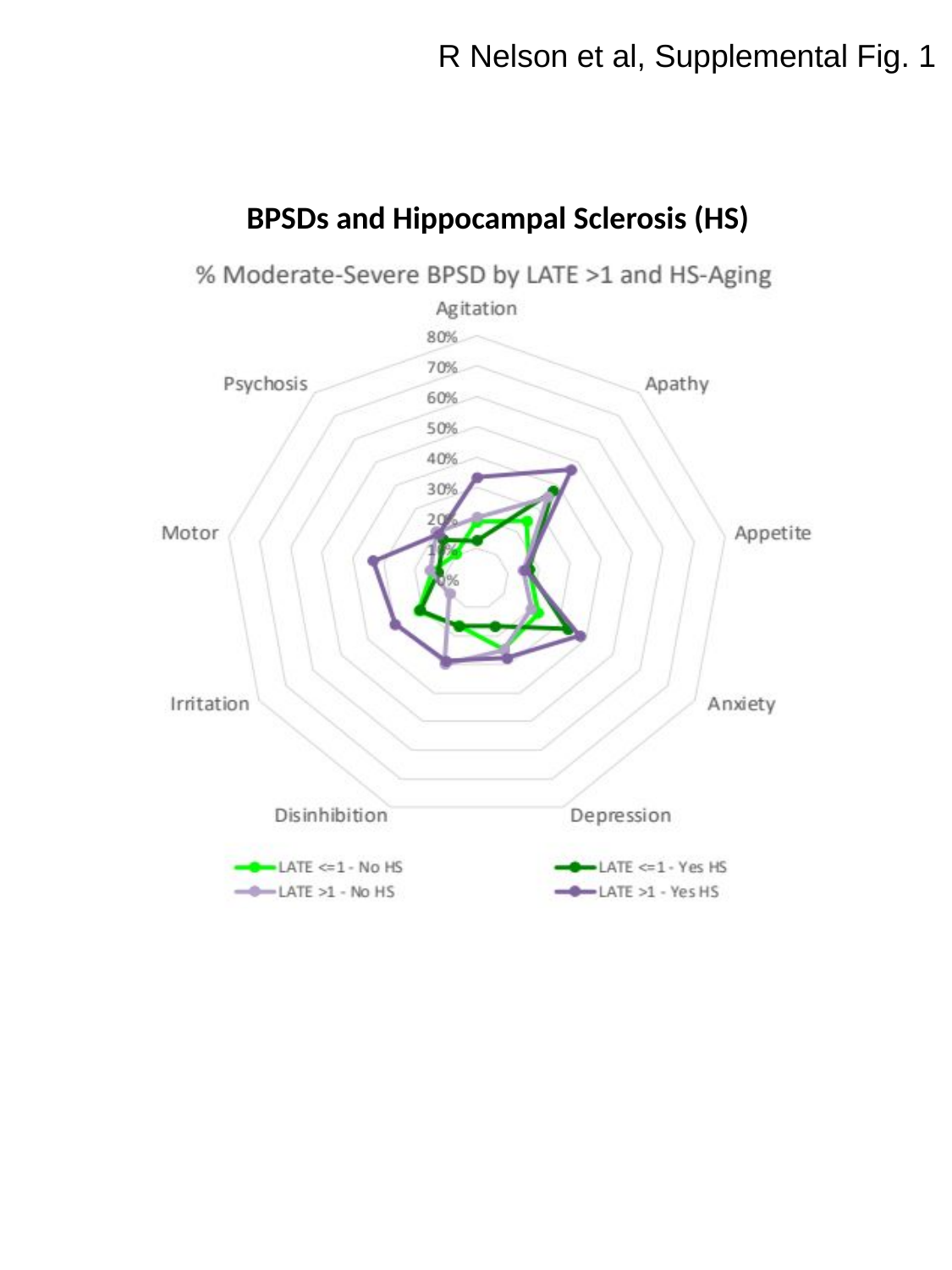

R Nelson et al, Supplemental Fig. 1
BPSDs and Hippocampal Sclerosis (HS)

## Slide 2
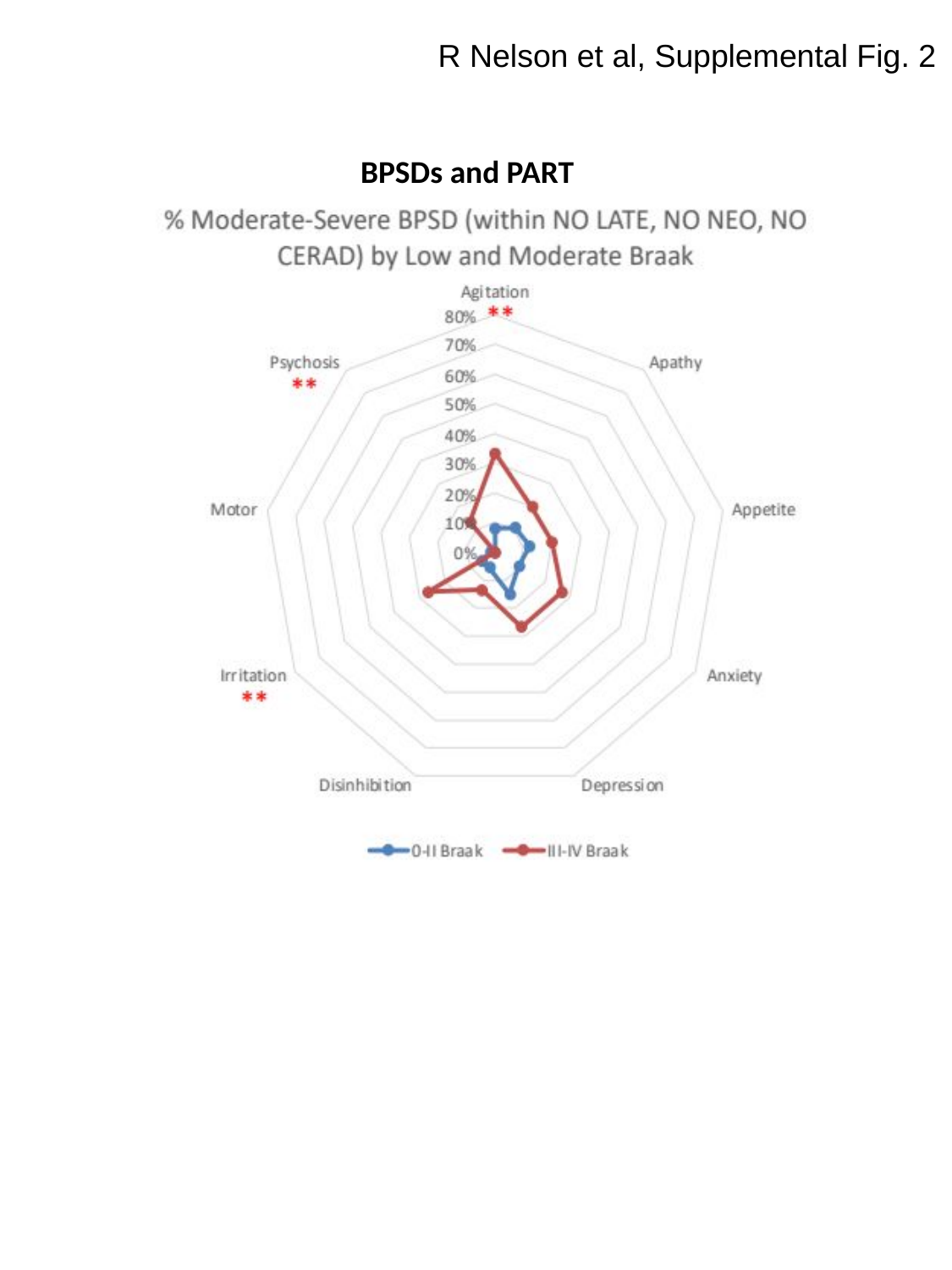

R Nelson et al, Supplemental Fig. 2
BPSDs and PART

## Slide 3
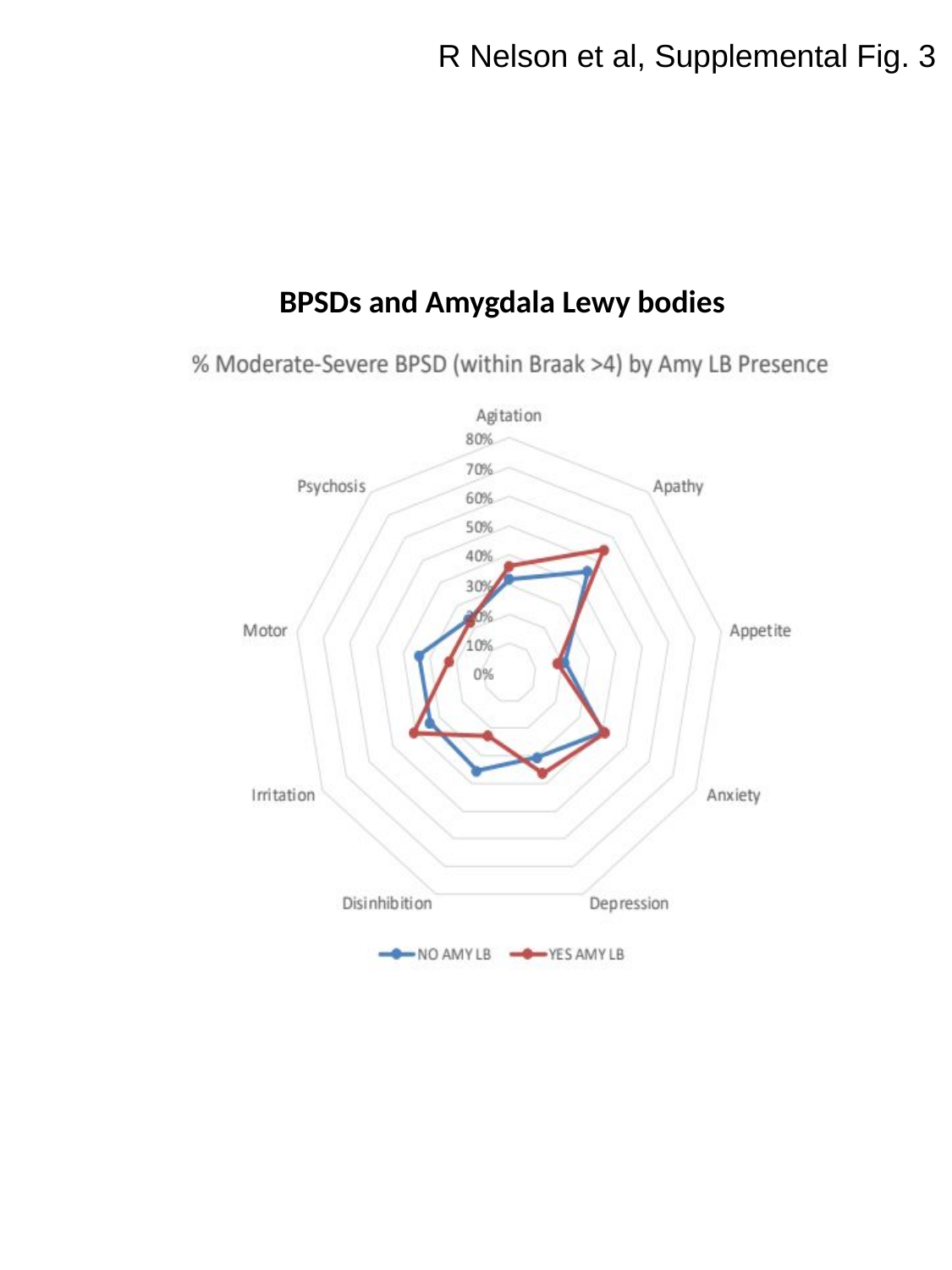

R Nelson et al, Supplemental Fig. 3
BPSDs and Amygdala Lewy bodies

## Slide 4
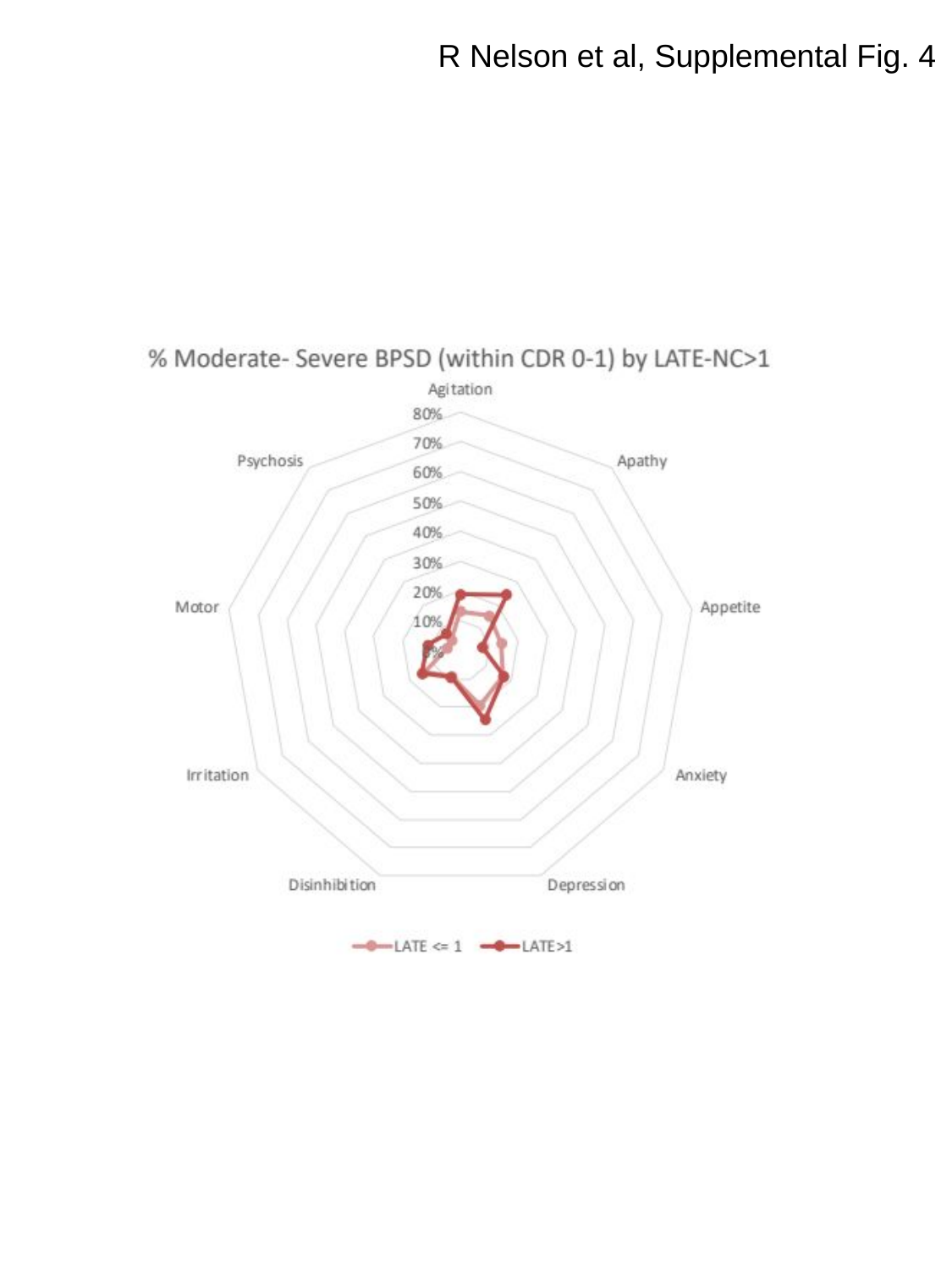

R Nelson et al, Supplemental Fig. 4

## Slide 5
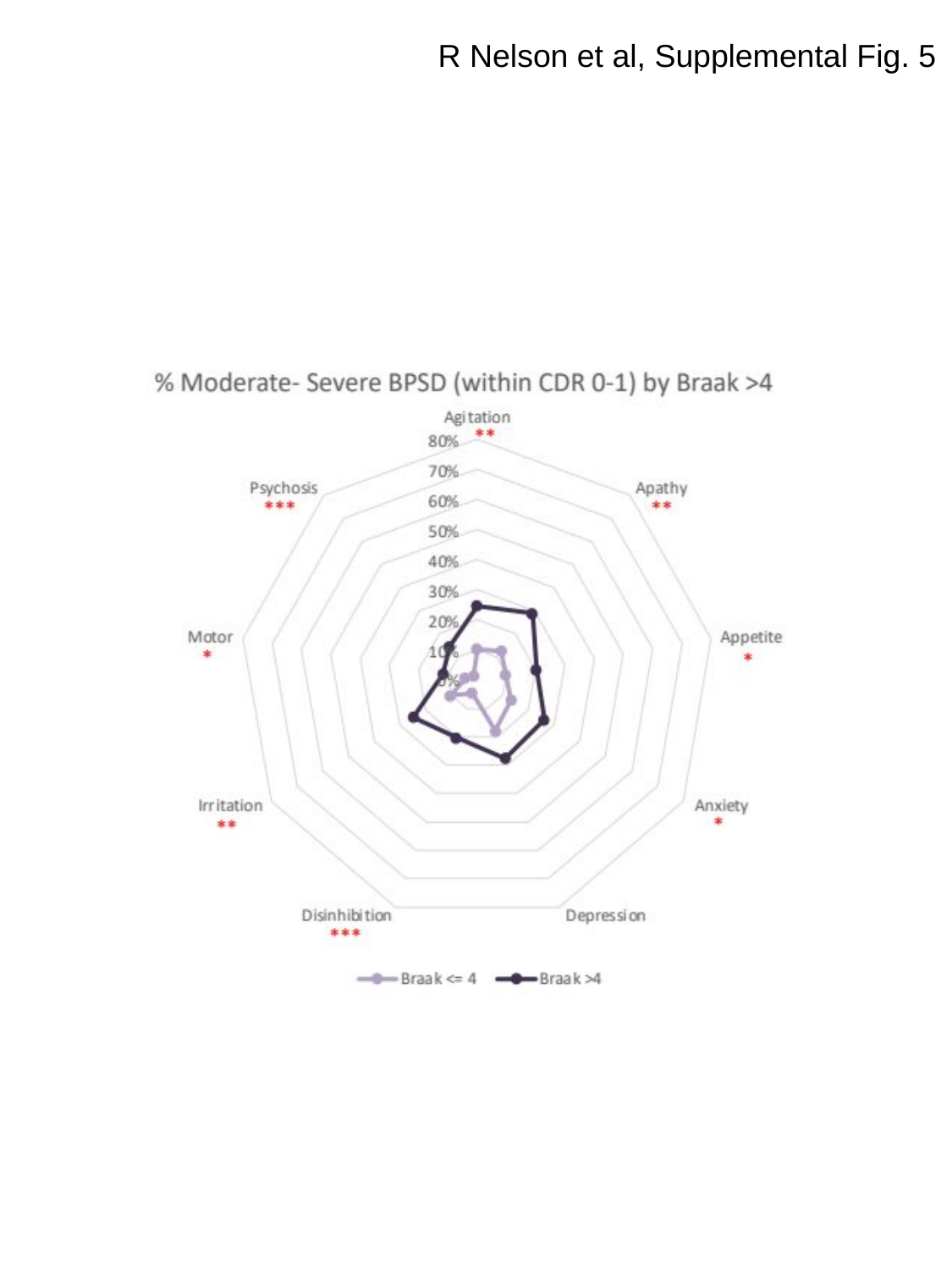

R Nelson et al, Supplemental Fig. 5

## Slide 6
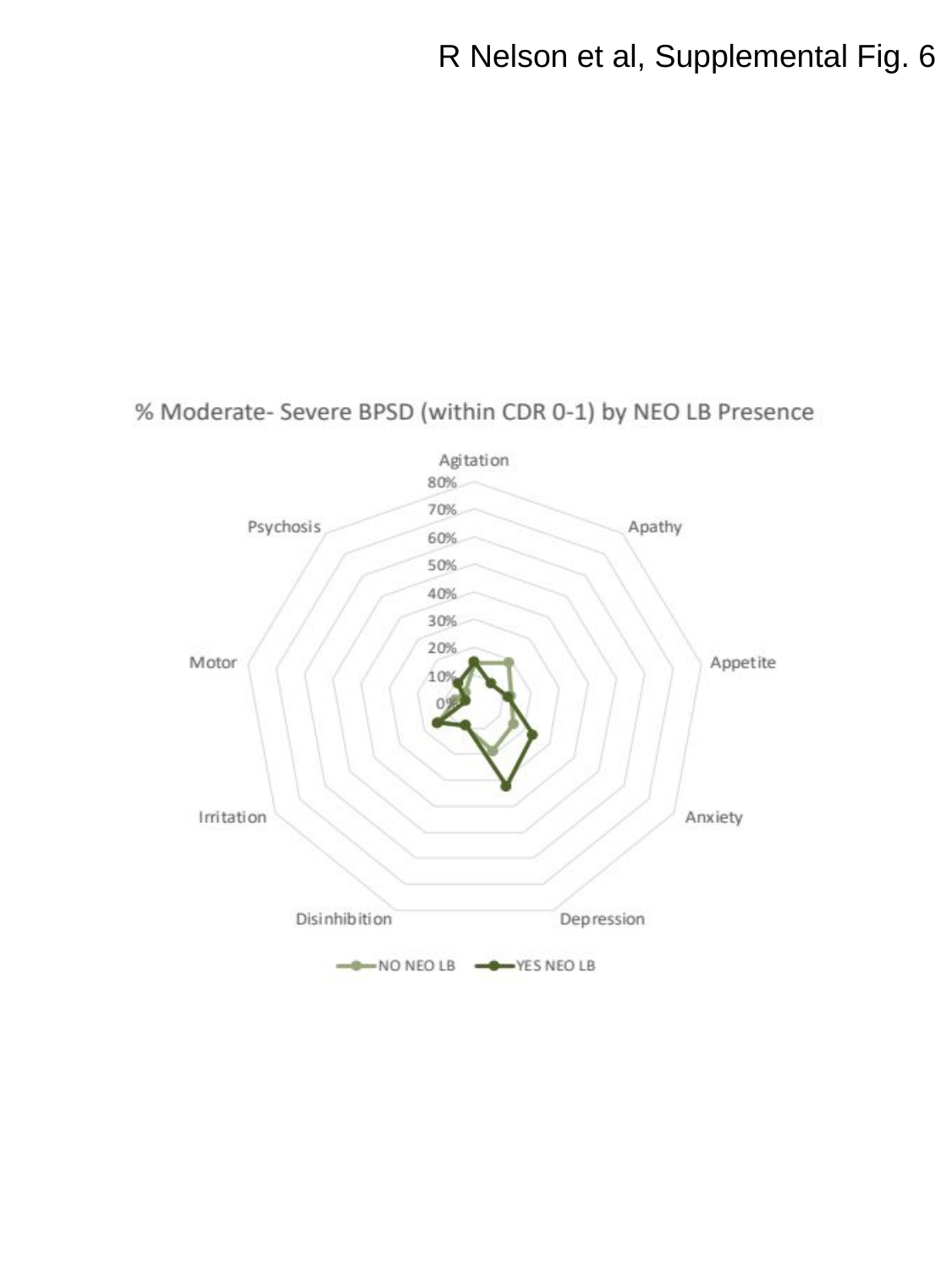

R Nelson et al, Supplemental Fig. 6
